# Orbital and intracranial *Nocardia farcinica* infection caused by trauma to the orbit: a case report

**DOI:** 10.1186/s12879-019-4605-z

**Published:** 2019-11-08

**Authors:** Anan Wang, Qihua Xu, Yaohua Wang, Hongfei Liao

**Affiliations:** Affiliated Eye Hospital of Nanchang University, 463 Bayi Road, Nanchang, 330000 Jiangxi China

**Keywords:** Antibiotic therapy, Endoscope, Local debridement, *Nocardia farcinica*, Orbital infection, Trauma

## Abstract

**Background:**

Localized `and disseminated *Nocardia farcinica* infection is frequently reported in immunocompromised patients. However, orbital nocardiosis is rare, and, to our knowledge, traumatic orbital nocardiosis that affects the brain has never been described. Here, we report a case of traumatic orbital and intracranial *N. farcinica* infection in an immunocompetent patient.

**Case presentation:**

A 35-year-old man, who was immunocompetent, to the best of our knowledge and as per the absence of immunodeficiency symptoms, with orbital trauma caused by the penetration of a rotten bamboo branch developed lesions in the orbit and brain. Subsequently, he underwent debridement and received broad-spectrum antibiotic therapy, but orbital infection occurred, with drainage of pus through the sinus tract. The patient then underwent endoscope-assisted local debridement. Bacterial culture of the sinusal pus was positive for *N. farcinica*, and a combined intracranial infection had developed. The disease was treated effectively by trimethoprim-sulfamethoxazole and ceftriaxone sodium therapy. The patient remained infection free and without complications at the 14-month follow-up.

**Conclusions:**

Traumatic orbital and intracranial infection caused by *N. farcinica* is a rare infectious disease, and atypical presentations easily lead to misdiagnosis. When a patient presents with an atypical orbital infection that is unresponsive to empirical broad-spectrum antibiotics, along with suspicious neurologic symptoms, *Nocardia* infection should be considered. Identification by bacterial culture is the gold standard. Complete local debridement and appropriate antibiotic treatment are keys to the treatment of the disease.

## Background

*Nocardia farcinica* infection is rare and often occurs in immunocompromised patients and is especially attributable to the respiratory tract or traumatic wounds [1]. *N. farcinica* infection caused by injuries occurs most commonly in the limbs and skin [2]. Orbital infection due to orbital trauma is uncommon. To our knowledge, orbital nocardiosis with brain infection has never been described. Here, we report a case of orbital and intracranial *N. farcinica* infection caused by trauma to the orbit in an immunocompetent man.

## Case presentation

**Fig. 1:**
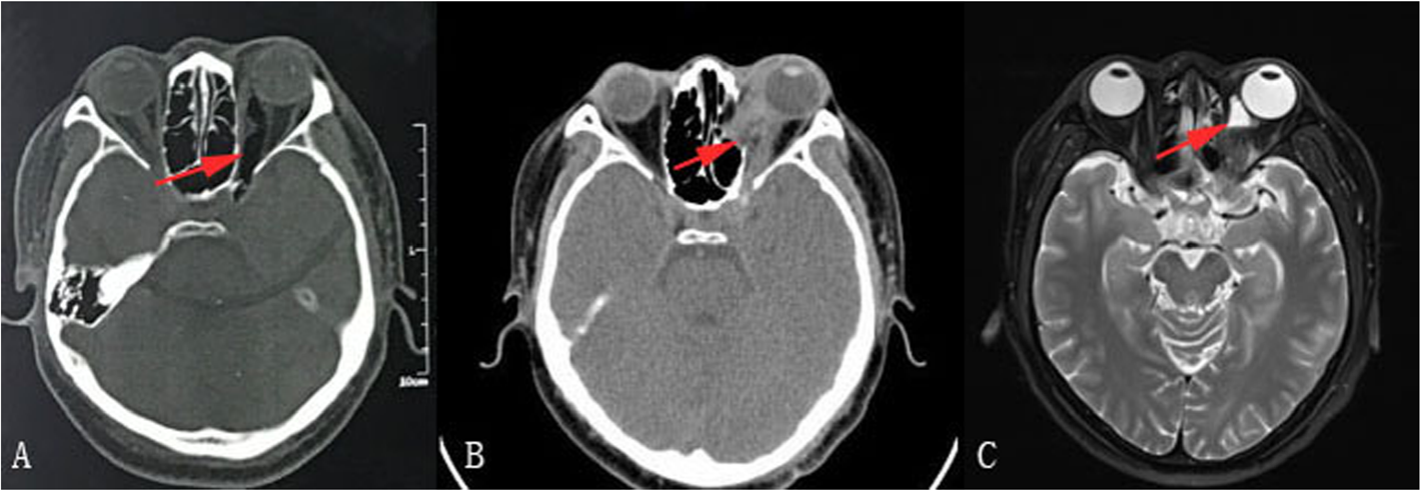
CT and MRI scans of the brain and orbit. **a** CT shows both orbit and brain lesions, as evidenced by decreased density in the orbit and brain. **b** CT shows an increase in the density in the orbit and a discontinuous medial wall of the orbital bone. **c** MRI sagittal T2 shows hyperintensity in the superonasal region of the left eye

A 35-year-old man was injured, when a rotten bamboo branch penetrated his left orbit while working in a mountainous area, and was immediately treated at a local hospital. The local hospital medical records showed a 2-cm irregular wound in the left upper margin of his eyelid, proptosis, ophthalmoplegia, impaired visual acuity, a dilated pupil, and absence of pupillary light reflex in his left eye. Computed tomography (CT) showed both orbit and brain lesions (Fig. 1a). The patient was immediately treated with antibiotics and steroids. Furthermore, due to the development of disease in his left eye, the patient underwent three operations, namely the removal of an intraorbital foreign body, debridement, and transnasal endoscopic orbital decompression, over the course of 2 weeks at the local hospital.

**Fig. 2:**
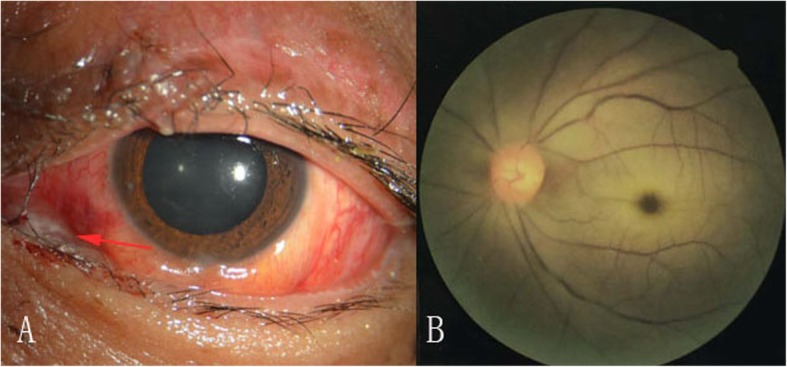
Anterior segment and fundus photography. **a** Anterior segment photography shows chemosis with inferonasal conjunctival purulent discharge and a dilated pupil. **b** Fundus photography shows retinal edema, narrow blood vessels, and macular cherry-red spot

The patient was transferred to our hospital without obvious improvement with the abovementioned treatments. CT showed proptosis, increased density in the orbit, and a discontinuous medial wall of the orbital bone in the left eye (Fig. 1b). Magnetic resonance imaging (MRI) showed hyperintensity in the superonasal region of the left eye (Fig. 1c). An ocular examination revealed the absence of light perception, orbital swelling, ptosis, proptosis, ophthalmoplegia, absence of pupillary light reflex, and purulent discharge from the inferonasal conjunctival sinus (Fig. 2a). Fundoscopic examination revealed retinal edema and macular cherry-red spot (Fig. 2b). No obvious abnormalities were found on a general examination, there was no increase in the C-reactive protein (0.8 mg/L, normal range 0–10 mg/L), other than abnormal laboratory exams including leukocytosis (22.62 × 10^9^/L, normal range 3.5–9.5 × 10^9^/L) and increased neutrophils (18.81 × 10^9^/L, normal range 1.8–6.3 × 10^9^/L).

**Fig. 3:**
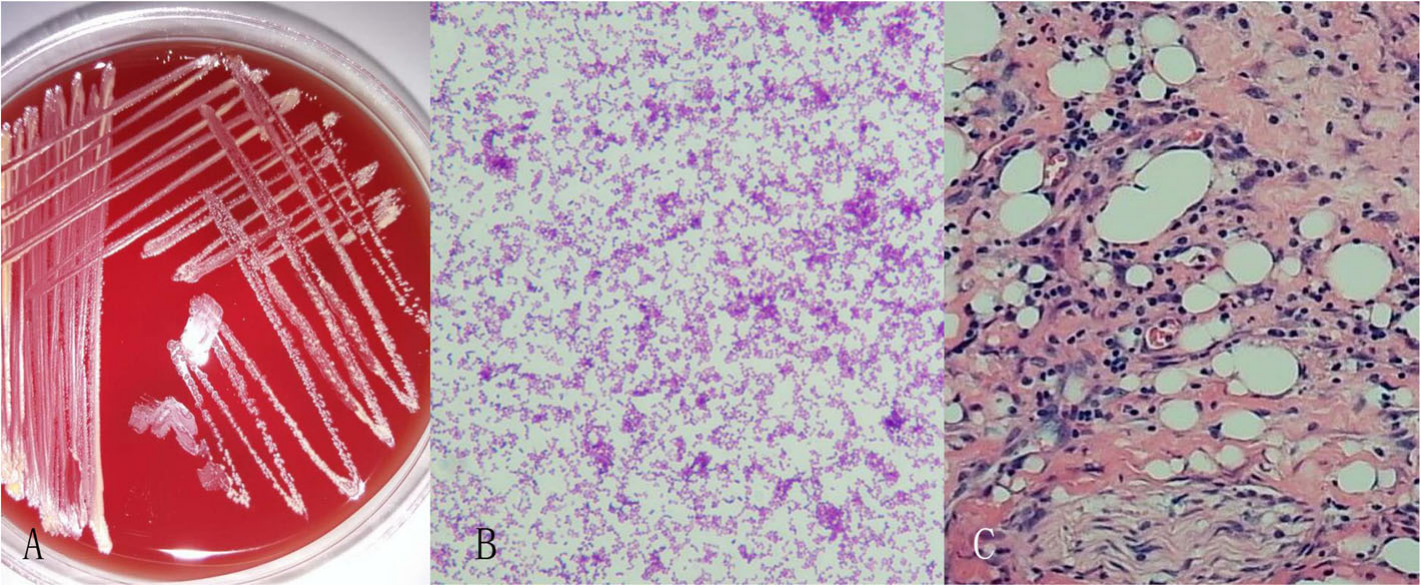
Bacterial culture results and histological assessment of the orbital abscess. **a** Bacterial culture shows white, unequal, wrinkling, and granular colonies. **b** Cultured *Nocardia farcinica* presents as gram-positive, thin, bacillary and coccoid forms (Gram’s staining, Original magnification × 1000). **c** Histological finding of the orbital abscess shows chronic putrid abscess formation (H&E staining, Original magnification × 100)

Pus from the conjunctival sinus was taken for culturing. Antibiotics such as ceftazidime (2 g every 12 h) and metronidazole (0.5 g every 12 h) were administered immediately. Five days after admission, the patient underwent endoscope-assisted orbitotomy. We made an incision on the sinus and exposed the deep orbital tissue through the sinus tract. To avoid the limitation of a small operating visual field, orbital abscesses and a few orbital foreign bodies were removed using an endoscope. Purulent fluid was obtained for repeated culture. Multiple incisional biopsies were performed and sent for histopathological examination. On the first postoperative day, *N. farcinica* was cultured from the pus of conjunctival sinus (Fig. 3 a-b). Histological examination of orbital tissues showed chronic pyogenic inflammation on the second postoperative day (Fig. 3c). The patient developed high fever and complained of headache. Laboratory examinations revealed moderate leukocytosis (16.93 × 10^9^/L, normal range 3.5–9.5 × 10^9^/L), increased neutrophils (14.3 × 10^9^/L, normal range 1.8–6.3 × 10^9^/L), and elevated C-reactive protein (30.77 mg/L, normal range 0–10 mg/L).

After 1 week of ceftazidime and metronidazole therapy and orbital surgical treatment, the patient was transferred to the infection department and immediately treated with oral trimethoprim-sulfamethoxazole (4 g every 12 h) and intravenous mannitol (25 g every 12 h). MRI of the head, chest, and abdomen showed no abnormalities. A lumbar puncture yielded clear cerebrospinal fluid with leukocytes 480/μL (normal range 0–10/μL, 45% neutrophils and 55% lymphocytes), glucose 1.98 mol/L (normal range 2.8–4.4 mol/L), chloride 118.2 mol/L (normal range 120–132 mol/L), protein 1046 mg/L (normal range 150–450 mol/L mg/L), and a positive Pandy’s test. Five days later, antibiotic therapy with 2 g ceftriaxone sodium every 12 h was initiated.

**Fig. 4:**
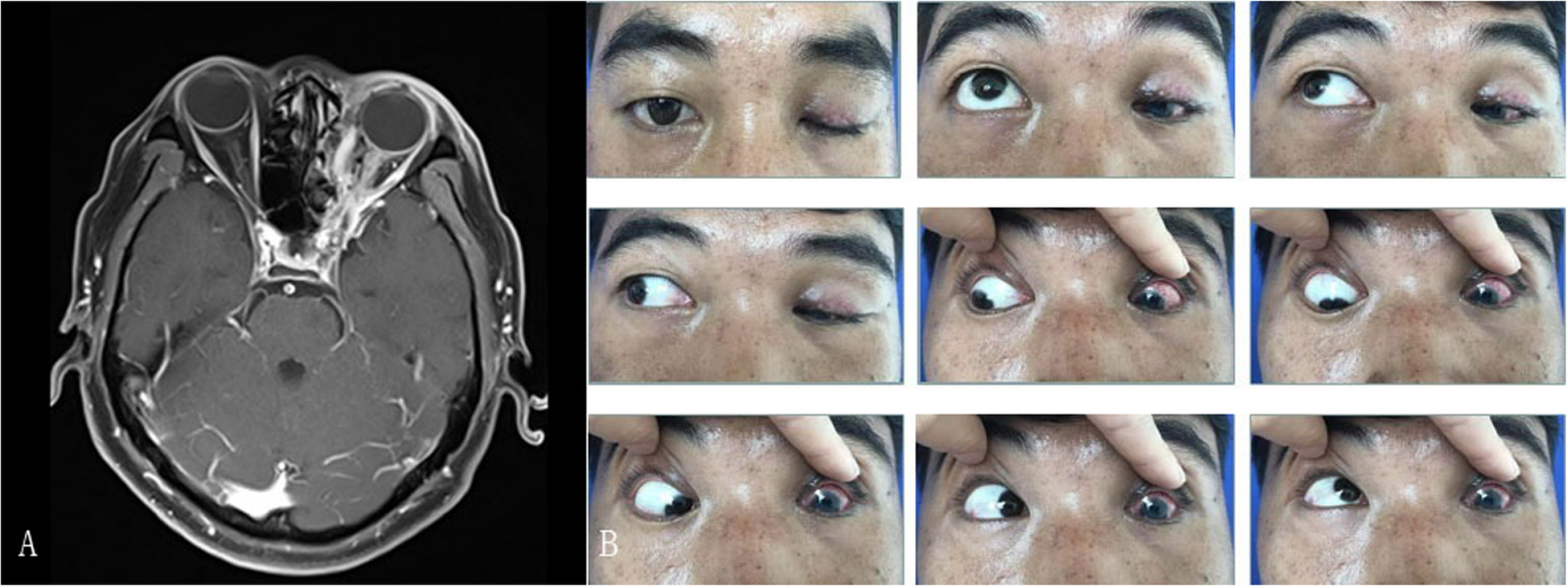
Orbital MRI scan and photographs. **a** MRI (enhanced) shows mild disordered structure of the left orbit. **b** Photographs show ptosis, ophthalmoplegia, and conjunctival congestion of the left eye

The patient responded well to the drug treatment with trimethoprime-sulfamethoxazole (4 g every 12 h) for 28 days, 20% mannitol (25 g every 12 h) for 19 days, and ceftriaxone sodium (2 g every 24 h) for 23 days, and a repeat MRI was performed 1 month postoperatively (Fig. 4a). However, blindness, ptosis, and ophthalmoplegia persisted (Fig. 4b). To prevent the recurrence of infection, the patient was advised to continue trimethoprim-sulfamethoxazole intake for 3 months. The dose in the first 2 months was 4 g every 12 h and subsequently, 3.2 g every 12 h for a month. The patient remained stable over a 14-month follow-up period.

## Discussion and conclusions

*N. farcinica* is an aerobic, gram-positive, filamentous, ubiquitous, soilborne, and weakly acid-fast bacteria [3, 4]. *N. farcinica* infections are usually acquired by direct inhalation of contaminated particles from soil or water; however, these infections are also reported to occur after traumatic injury [5, 6]. Misdiagnosis and mistreatment of *N. farcinica* infection can cause severe damage and even death, because *Nocardia* species can disseminate and are resistant to antibiotics.

Nocardiosis often affects immunocompromised individuals. The patient in this case had no obvious immunodeficiency and was infected due to traumatic orbital injury. Infection by direct orbital injury is rare, as most injury-mediated infections occur in the limbs and skin [2]. According to Torres et al., a literature review of nocardiosis showed that traumatic injuries accounted for only 10% of infections [1]. Another review showed that *N. farcinica* accounted for 5% of all nocardiosis [6]. Additionally, concurrent orbital and intracranial *N. farcinica* infections due to injury have not been previously reported.

Clinical manifestations of orbital infection usually involve periorbital edema, crepitus, ophthalmoplegia, exophthalmos, chemosis, and visual loss [2, 7]. The case we have reported here had no other specific features, and the symptoms mentioned above are similar to those for subacute local infection. However, the infection in our patient also involved the brain, and the patient experienced high fever and headache. Nocardiosis often disseminates hematogenously to distant organs, such as the lungs, kidneys, joints, and bones [1]. In our patient, the infection did not spread to other organs, possibly because he was young and immunocompetent.

Thus far, isolation and identification of *Nocardia* strains is the only reliable diagnostic method. *Nocardia* species are strictly aerobic and grow slowly at 35 °C in standard culture medium. Hence, it is important to inform the microbiological laboratory that nocardiosis with soil/environmentally contaminated penetrating traumas should be considered, even among immunocompetent patients, to facilitate the identification of *Nocardia* species. *N. farcinica* grew from cultures of conjunctival pus samples from our patient. Bacteria were not detected in cultures from other body fluids, including orbital abscess secretions, cerebrospinal fluid, and blood, most likely due to the antibiotic therapy. Microscopic examination of *Nocardia* revealed that these are gram-positive, thin, branching, filamentous, bacillary, or coccoid bacteria [8]. Identification procedures include biochemical, chemotaxonomic, serological, antimicrobial susceptibility testing, and molecular methods. Molecular techniques are more rapid and precise than other methods [8]. In our case, *N. farcinica* presented as bacillary or coccoid forms, and bacterial identification was performed using an emerging molecular technique, namely matrix-assisted laser desorption ionization-time of flight mass spectrometry, which is a rapid, sensitive, and economical method for identifying and diagnosing microbial infections [9].

Complete local debridement and appropriate antibiotic therapy are important in the treatment of *Nocardia* infections [10]. The infectious lesion was located deep within the orbit, making its exposure difficult. As such, endoscope-assisted debridement was important for excising the abscesses efficiently and accurately. Appropriate antibiotic administration is another critical factor to treat nocardiosis, and susceptibility testing is of vital importance as the susceptibility pattern of *Nocardia* species is highly variable. In our case, drug susceptibility test was not performed, because this was the first case of nocardia infection in our hospital, and paper diffusion method reference standard for the drug susceptibility test on Clinical and Laboratory Standards Institute is not available. However, patients must undergo antibiotic therapy immediately after the diagnosis of *N. farcinica* infection. Trimethoprim-sulfamethoxazole is the first choice for the treatment of *N. farcinica* infections before obtaining the susceptibility-test result [1, 4]. Empiric combination therapy of trimethoprim-sulfamethoxazole and ceftriaxone is also recommended [6]. The therapy needs to be continued for several months due to the high possibility of infection recurrence, which depends on the immune status of the patient. If the central nervous system is affected, the therapy should be continued at least for 6 months. In our case, the patient was immunocompetent and was treated with antibiotic therapy for 3 months, and there was no recurrence of infection at 14-month follow-up.

In conclusion, due to the low incidence of orbital *Nocardia* infections, these are not well characterized and are often not considered in an initial diagnosis. When a patient presents with an atypical orbital infection that is unresponsive to empirical broad-spectrum antibiotics, along with suspicious neurologic symptoms, *Nocardia* infection should be considered. Misdiagnosis and inappropriate therapy may result in serious consequences. The present case also highlights the clinical features, diagnosis, and novel management of *Nocardia* infection using endoscope-assisted local debridement. Appropriate antibiotic treatment based on susceptibility testing is another critical component of the treatment for *N. farcinica* infections.

## Data Availability

Not applicable.

## Abbreviations

CT: Computed tomography
MRI: Magnetic resonance imaging
